# Asthma-Associated Emergency Department Visits During the Canadian Wildfire Smoke Episodes — United States, April– August 2023

**DOI:** 10.15585/mmwr.mm7234a5

**Published:** 2023-08-25

**Authors:** Cristin E. McArdle, Tia C. Dowling, Kelly Carey, Jourdan DeVies, Dylan Johns, Abigail L. Gates, Zachary Stein, Katharina L. van Santen, Lakshmi Radhakrishnan, Aaron Kite-Powell, Karl Soetebier, Jason D. Sacks, Kanta Sircar, Kathleen P. Hartnett, Maria C. Mirabelli

**Affiliations:** ^1^Epidemic Intelligence Service, CDC; ^2^Division of Environmental Health Science and Practice, National Center for Environmental Health, CDC; ^3^Oak Ridge Institute for Science and Education, Oak Ridge, Tennessee; ^4^Detect and Monitor Division, Office of Public Health Data, Surveillance, and Technology, CDC; ^5^ICF International, Reston, Virginia; ^6^Center for Public Health and Environmental Assessment, Office of Research and Development, Environmental Protection Agency, Research Triangle Park, North Carolina.

**Summary**

**What is already known about this topic?**

As wildfires and wildfire smoke increase across the United States, symptoms of wildfire smoke exposure are of increasing public health concern.

**What is added by this report?**

Emergency department visits for asthma were 17% higher than expected during 19 days of wildfire smoke that occurred during April–August 2023.

**What are the implications for public health practice?**

Changes in asthma-associated emergency department visits during and after periods of wildfire smoke can be used by public health communicators, clinicians, policymakers, and the public to monitor and reduce exposure to wildfire smoke for persons with asthma.

## Abstract

During April 30–August 4, 2023, smoke originating from wildfires in Canada affected most of the contiguous United States. CDC used National Syndromic Surveillance Program data to assess numbers and percentages of asthma-associated emergency department (ED) visits on days with wildfire smoke, compared with days without wildfire smoke. Wildfire smoke days were defined as days when concentrations of particulate matter (particles generally ≤2.5 *μ*m in aerodynamic diameter) (PM_2.5_) triggered an Air Quality Index ≥101, corresponding to the air quality categorization, “Unhealthy for Sensitive Groups.” Changes in asthma-associated ED visits were assessed across U.S. Department of Health and Human Services regions and by age. Overall, asthma-associated ED visits were 17% higher than expected during the 19 days with wildfire smoke that occurred during the study period; larger increases were observed in regions that experienced higher numbers of continuous wildfire smoke days and among persons aged 5–17 and 18–64 years. These results can help guide emergency response planning and public health communication strategies, especially in U.S. regions where wildfire smoke exposure was previously uncommon.

## Introduction

Millions of U.S. adults and children have been exposed to wildfire smoke* caused by smoke plumes originating from wildfires in Canada that began in April 2023 (*1*). Wildfire smoke is a complex mixture containing gases and particles, where particulate matter (particles generally ≤2.5 *μ*m in aerodynamic diameter) (PM_2.5_) is the pollutant of most health concern because it can exacerbate existing cardiovascular, metabolic, and respiratory conditions and thus lead to increased emergency department (ED) visits and hospitalizations based on day-to-day changes in wildfire smoke exposure (*2*–*4*). However, little is known about the health implications of prolonged episodes of high concentrations of wildfire smoke, such as those experienced during the recent wildfires in Canada. As a result, rapid assessment of related health impacts is needed to guide risk communications and reduce exposures and health effects attributed to wildfire smoke.

## Methods

Wildfire smoke event days are defined at the U.S. Department of Health and Human Services (HHS) region^†^ level when at least one Environmental Protection Agency (EPA) air quality monitor^§^ in the region measures ambient 24-hour average PM_2.5_ concentrations ≥35.5 *μ*g/m^3^ (*5*), corresponding to the EPA Air Quality Index (AQI)^¶^ value of 101. AQI of 101 was selected because AQI ≥101 is the threshold for categorizing air quality as unhealthy. As the AQI increases, air quality becomes increasingly unhealthy (i.e., “Unhealthy for Sensitive Groups” [AQI *=* 101–150], “Unhealthy” [AQI *=* 150–200], “Very Unhealthy” [AQI *=* 201–300], and “Hazardous” [AQI ≥301]).

CDC analyzed data from the National Syndromic Surveillance Program (NSSP). NSSP collects data from approximately 6,000 EDs, representing 76% of all eligible facilities in the United States; 4,317 facilities, representing 85% of all NSSP facilities, were included in this analysis (*6*). Asthma-associated ED visits were defined as those with mention of asthma as the chief complaint for the ED visit.

Observed daily numbers and percentages of asthma-associated ED visits during April 30–August 4, 2023, were compared with expected numbers and percentages, stratified by HHS region and age group (0–4, 5–17, 18–64, and ≥65 years). Observed visits were defined as the number of visits reported to NSSP on a given day and expected visits were calculated using anomaly detection algorithms** (*6*) applied to the preceding 30 days of ED visits, excluding the most recent 2 days. Excess asthma-associated ED visits were calculated as the sum of observed visits minus the sum of expected visits for days with wildfire smoke exposure. Visit anomalies (i.e., higher-than-expected numbers of asthma-associated ED visits) were detected when either the number or percentage of asthma-associated ED visits was significantly higher than expected. Student *t*-tests were used to derive p-values, and p<0.05 was considered statistically significant. NSSP data were extracted from the Electronic Surveillance System for the Early Notification of Community-Based Epidemics (ESSENCE) via the Rnssp package^††^ and analyzed using R software (version 4.3.1; R Foundation). This activity was reviewed by CDC and was conducted consistent with applicable federal law and CDC policy.^§§^

## Results

**FIGURE 1:**
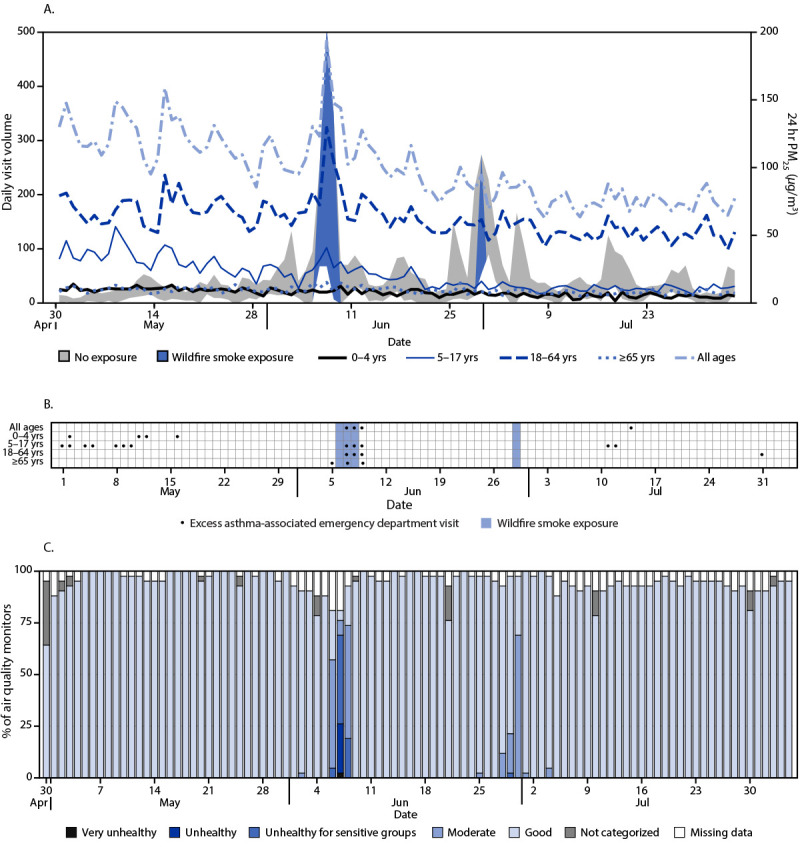
Trends in asthma-associated emergency department visits (A), excess asthma-associated emergency department visit detection (B), and the percentage of air quality monitors* reporting concentrations of fine particulate matter ≤2.5 *μ*m in aerodynamic diameter indicative of wildfire smoke (C), by day — U.S. Department of Health and Human Services Region 2,^†^ April 30, 2023–August 4, 2023^§^ **Abbreviation**: PM_2.5_ = particulate matter with aerodynamic diameter ≤2.5 *μ*m. * https://www.airnow.gov/aqi/aqi-basics/ ^†^ New Jersey and New York (Puerto Rico and U.S. Virgin Islands do not report data to the National Syndromic Surveillance Program). ^§^ A wildfire smoke exposure day occurs when at least one air quality monitor in the region reports PM_2.5_ concentrations corresponding to an Air Quality Index of ≥101.

**FIGURE 2:**
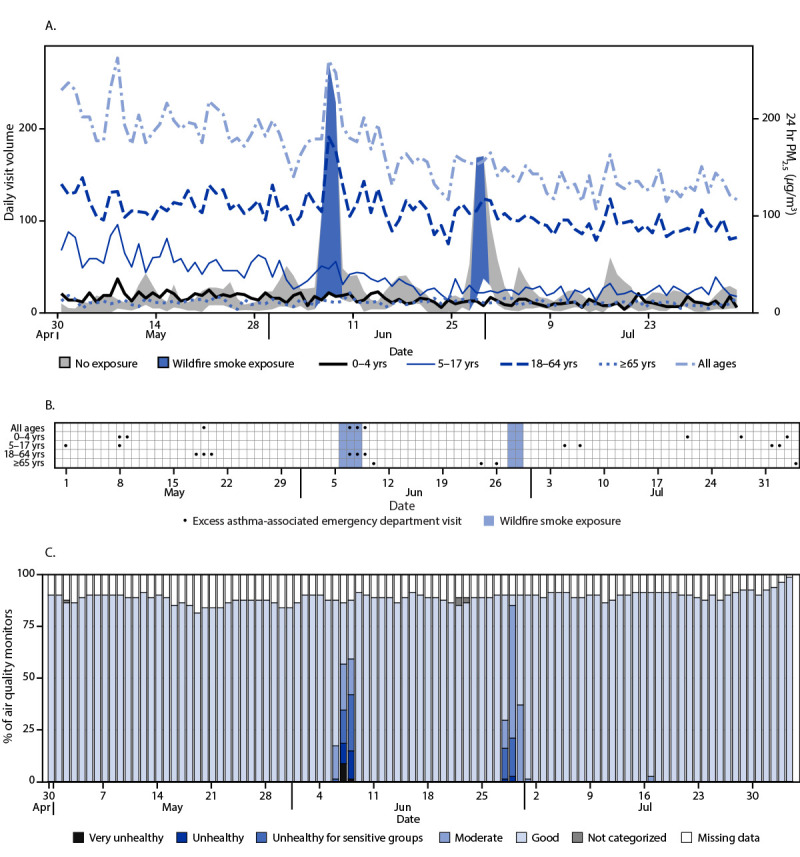
Trends in asthma-associated emergency department visits (A), excess asthma-associated emergency department visit detection (B), and the percentage of air quality monitors* reporting concentrations of fine particulate matter ≤2.5 *μ*m in aerodynamic diameter indicative of wildfire smoke (C), by day — U.S. Department of Health and Human Services Region 3,^†^ April 30, 2023–August 4, 2023^§^ **Abbreviation:** PM_2.5_ = particulate matter with aerodynamic diameter ≤2.5 *μ*m. * https://www.airnow.gov/aqi/aqi-basics/ ^†^ Delaware, District of Columbia, Maryland, Pennsylvania, Virginia, and West Virginia. ^§^ A wildfire smoke exposure day occurs when at least one air quality monitor in the region reports PM_2.5_ concentrations corresponding to an Air Quality Index of ≥101.

**FIGURE 3:**
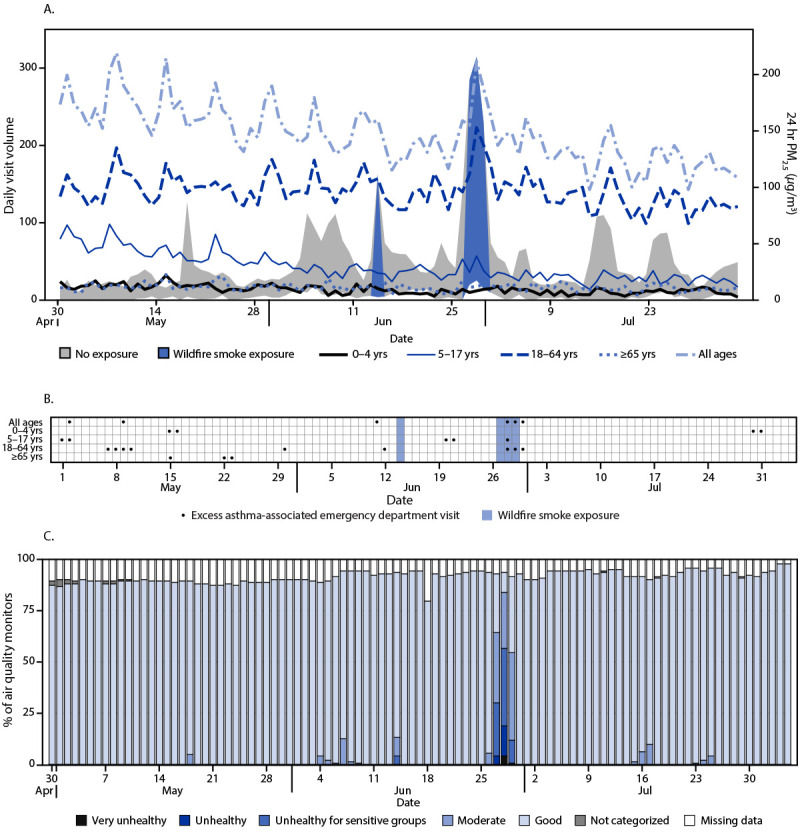
Trends in asthma-associated emergency department visits (A), excess asthma-associated emergency department visit detection (B), and the percentage of air quality monitors* reporting concentrations of fine particulate matter ≤2.5 *μ*m in aerodynamic diameter indicative of wildfire smoke (C), by day — U.S. Department of Health and Human Services Region 5,^†^ April 30, 2023–August 4, 2023^§^ **Abbreviation**: PM_2.5_ = particulate matter with aerodynamic diameter ≤2.5 *μ*m. * https://www.airnow.gov/aqi/aqi-basics/ ^†^ Illinois, Indiana, Michigan, Minnesota, Ohio, and Wisconsin. ^§^ A wildfire smoke exposure day occurs when at least one air quality monitor in the region reports PM_2.5_ concentrations corresponding to an Air Quality Index of ≥101.

During days of wildfire smoke occurring during April 30–August 4, 2023, overall observed asthma-associated ED visits^¶¶^ were 17% higher than expected among all age groups and HHS regions. Increased (excess) asthma-associated ED visits were detected more commonly on days with a higher percentage of air quality monitors reporting PM_2.5_ concentrations indicative of a wildfire smoke day (Supplementary Table, https://stacks.cdc.gov/view/cdc/132183). Specifically, Region 2 (Figure 1), Region 3 (Figure 2), and Region 5 (Figure 3) experienced the most wildfire smoke days with the highest reported PM_2.5_ concentrations, the highest percentages of air quality monitors detecting wildfire smoke, and the highest number of excess asthma-associated ED visits.

Region 3 experienced 5 wildfire smoke event days, the highest total amount for any region with more than 1% of air quality monitors reporting AQI ≥101, with a maximum 24-hour average PM_2.5_ concentration of 259 *μ*g/m^3^, and Region 2 and Region 5 experienced a total of 4 wildfire smoke event days, with each reporting a maximum 24-hour average PM_2.5_ concentration of 204 *μ*g/m^3^ and 216 *μ*g/m^3^, respectively. Within the identified smoke event days, the percentages of air quality monitors reporting wildfire smoke by HHS region ranged from 0.5%–69.0% (Supplementary Table, https://stacks.cdc.gov/view/cdc/132183).

Region 2 experienced the largest increase in asthma-associated ED visits. During June 6–8, higher-than-expected asthma-associated ED visits occurred for all age groups on 2 days, representing 364 excess visits, and among patients aged 5–17 (2 days, 123 excess visits), 18–64 (2 days, 251 excess visits), and ≥65 years (2 days, 12 excess visits) (Figure 1) (Supplementary Table, https://stacks.cdc.gov/view/cdc/132183). On another day (June 29), wildfire smoke was detected at 2.4% of stations, but no days of higher-than-expected asthma-associated ED visits were detected in any age group (Figure 1).

In Region 3, during June 6–8, 1 day of higher-than-expected asthma-associated ED visits was observed among all age groups combined (179 excess visits), and 2 days of higher-than-expected visits were observed among patients aged 18–64 years (128 excess visits). During June 28–29, no higher-than-expected asthma-associated ED visits were observed (Figure 2). In Region 5 during June 27–29, 1 day of higher-than-expected asthma-associated ED visits was observed among all age groups (172 excess visits) and among persons aged 5–17 years (14 excess visits); among persons aged 18–64 years, 2 days of higher than expected asthma-associated ED visits were observed (155 excess visits).

Regions 1, 4, and 9 each experienced 1 day of wildfire smoke and, within these regions, higher-than-expected asthma-associated ED visits were only observed in Region 4. Region 7 experienced 4 days of wildfire smoke, but asthma-associated ED visits were not increased. In Region 8, 3 wildfire smoke days and 1 day of higher-than-expected asthma-associated ED visits occurred among persons aged 18–64 years, representing 18 excess visits. In Region 10, 4 wildfire smoke days with less than 1% of air quality monitors reporting AQI ≥101 had higher-than-expected asthma-associated ED visits representing 14 excess visits among persons aged 18–64 years. 

## Discussion

During 2023, wildfire smoke traveled hundreds of miles and affected communities resulting in multijurisdictional emergencies, air quality alerts, and significant increases in asthma-associated ED visits. Wildfire smoke had affected all HHS regions except Region 6 during April 30–August 4, 2023, resulting in ≥1 day of wildfire smoke. Increases in asthma-associated ED visits occurring during days of wildfire smoke highlight the need to reduce wildfire smoke exposure during such events and wildfire smoke–related morbidity across all age groups.

Asthma-associated ED visits increased in response to regional wildfire smoke patterns and when a higher percentage of air quality monitors reported AQI values ≥101 (PM_2.5_ ≥35.5 *μ*g/m^3^) indicative of more wildfire smoke. Higher-than-expected asthma-associated ED visits were observed among persons of all ages and those aged 5–17, 18–64, and ≥65 years but were most common among persons aged 18–64 years. Information was not available about the extent to which patients with asthma were able to follow exposure reduction measures during periods of high PM_2.5_ concentration. Asthma-associated ED visit anomalies, which represent higher-than-expected visits, were also detected on days without wildfire smoke. These anomalies were primarily among persons aged <5 years and 5–17 years and during the first one half of the study period.

Jurisdictions interested in using syndromic surveillance to monitor the public health implications of wildfire smoke might consider using asthma as an initial indicator to develop strategies to reduce exacerbations and reach populations at increased risk for both exposure and adverse health effects. Expanded monitoring of health conditions, including cardiopulmonary-related ED visits, might also improve understanding of the severity of the impact of wildfire smoke on health outcomes and amplify prevention efforts to reduce these exacerbations.

### Limitations

The findings in this report are subject to at least four limitations. First, AQI ≥101 occurred during the period of wildfires and the wildfire smoke plumes, but this report cannot directly attribute the increase in AQI to wildfires in Canada. Second, NSSP data are not nationally representative, and participation varies by HHS region. This report is aggregated by HHS region level and might not reflect subregional patterns of wildfire smoke health effects, especially in areas where air quality monitors and facilities do not have the same geographic distribution within HHS regions. Third, NSSP data contain information on persons who seek care through an emergency setting only and do not capture asthma-related visits through other health care settings (e.g., primary care and urgent care), which might underestimate the incidence of wildfire smoke–related health effects if those experiencing adverse health effects did not seek emergency care. Finally, wildfire smoke days were defined using AQI ≥101, which might not fully capture increases in PM_2.5_ attributed to wildfire smoke, specifically in areas with low PM_2.5_ concentrations where sharp increases can still result in AQI <101.

### Public Health Implications

The risk of wildfire smoke exposure is increasing because of climate change, land management practice, and growth of wildland-urban interface areas, particularly in locations that have not historically experienced wildfire smoke (*7*). Syndromic surveillance data identified excess asthma-associated ED visits related to wildfire smoke and serve as some of the earliest available detection indicators. Community preparedness and appropriate and prompt response are crucial to reduce wildfire smoke exposure and morbidity. Recommended actions include assessing a possible health care utilization surge related to wildfire smoke exposure. Clinicians can consider counseling patients on protective measures (e.g., awareness of current and predicted air quality levels, staying indoors, using air filtration, and using properly fitted N95 respirators when outdoors), especially among persons with asthma, chronic obstructive pulmonary disease, cardiovascular disease, or children, older adults, and pregnant persons (*8*). Additional guidance to protect from wildfire smoke can be found online (*9*) and by using AirNow’s Fire and Smoke Map, the AirNow app, or by listening to the Emergency Alert System and the National Oceanic and Atmospheric Administration’s Weather Radio to monitor wildfire smoke levels. The findings from this report provide actionable information to identify and engage in wildfire smoke preparedness and risk communications to meet the needs of populations at highest risk for wildfire smoke–related adverse health effects.

## Footnotes

* Approximation based on U.S. Census Bureau estimated decennial population distribution by U.S. Department of Health and Human Services region and Environmental Protection Agency monitors meeting at least one measured 24-hour average concentration ≥35.5 *μ*g/m^3^ for PM_2.5_ during April 30–August 4, 2023.

† Puerto Rico and U.S. Virgin Islands currently do not report data to the National Syndromic Surveillance Program. https://www.hhs.gov/about/agencies/iea/regional-offices/index.html

§ PM_2.5_ values are reported at air quality monitors and aggregated across 24-hour periods. These air quality monitor-level data are used at the HHS regional level, with the maximum and minimum air quality monitor daily PM_2.5_ values. Air quality monitors from all 50 states and District of Columbia are included. For the given period, 971–1,012 air quality monitors were reporting on a given day. Air quality monitors can report negative numbers or zero values. Air quality monitors not reporting on a given day were not categorized. Consistent reporting of air quality measures was reported during the study period with more than 76% of air quality monitors in each region reporting daily.

¶
https://www.airnow.gov/

** The anomaly detection method is automated to alternate between adaptive multiple linear regression and exponentially weighted moving average (EWMA) based on baseline data. When the regression model does not fit the baseline data well based on adjusted R-squared values (adjusted R-squared <0.60), then EWMA is used. Adaptive multiple linear regression fits a model to a baseline of 28 days and forecasts a predicted value 3 days after the last day of the baseline. The model adjusts for linear trends, day-of-week effects, and holidays. The predicted value is compared with the observed value and divided by the SE of prediction. In EWMA, weighted averages of recent data are compared with the average of the 28-day baseline and divided by the SD. When the p-value resulting from the Student’s *t*-test applied to the test statistic is <0.05, the data point is classified as an anomaly.

†† https://github.com/CDCgov/Rnssp

§§ 45 C.F.R. part 46.102(l)(2), 21 C.F.R. part 56; 42 U.S.C. Sect. 241(d); 5 U.S.C. Sect. 552a; 44 U.S.C. Sect. 3501 et seq.

¶¶ Calculated as the percentage with no age stratification for each HHS region during the exposure days using the equation [(excess ED visits / expected ED visit) x 100]. The percentage therefore represents the equation {[(observed ED visits − expected ED visits for exposure days) / expected ED visits] x 100}.
